# Influence factors and prediction model of enstrophy dissipation from the tip leakage vortex in a multiphase pump

**DOI:** 10.1038/s41598-022-20380-8

**Published:** 2022-09-26

**Authors:** Zekui Shu, Guangtai Shi, Xin Yao, Guodong Sun, Sijia Tao

**Affiliations:** 1 Key Laboratory of Fluid Machinery and Engineering, Xihua University, Chengdu, 610039 Sichuan China; 2 State Key Laboratory of Hydroscience and Engineering and Department of Energy and Power Engineering, Tsinghua University, Beijing, 100084 China; 3 Key Laboratory of Fluid and Power Machinery, (Xihua University), Ministry of Education, Chengdu, 610039 China

**Keywords:** Mechanical engineering, Fossil fuels, Applied physics, Fluid dynamics

## Abstract

In a multiphase pump, tip clearance is the required distance between the blade tip and the pump body wall of the impeller, forming tip leakage vortex (TLV), causing unstable flow and energy dissipation. In the present work, the enstrophy dissipation theory is innovatively applied to quantitatively study the energy dissipation of the TLV. The flow rate, tip clearance, and inlet gas void fraction (*IGVF*) play a crucial role in affecting the enstrophy dissipation of the TLV. The results show that increasing flow rate, tip clearance, and *IGVF* significantly exacerbate the TLV pattern and raise the TLV scale, which gradually raises volume enstrophy dissipation and decreases wall enstrophy dissipation. As the flow rate increases, the separation angle between the primary TLV trajectory and the blade gradually decreases, and widely dispersing the enstrophy dissipation near the shroud. However, as the tip clearance increases, the tip separated vortex scale increases and extends to the suction surface, raising the velocity gradient. Besides, as the *IGVF* increases, the secondary TLV develops from a continuous sheet vortex to a scattered strip vortex, increasing the significantly increasing the enstrophy dissipation. Considering the flow rate, tip clearance, and *IGVF* as independent variables, simple and multiple nonlinear regression models have the ability to predict the enstrophy dissipation of the TLV accurately.

## Introduction

The consumption of oil and gas resources accounts for up to 80% of the energy used to power global social production. After many years of exploitation and use, the onshore energy reserves are insufficient to meet future modern industrial production needs^1,2^. Experts all over the world have shifted their attention to the rich oil and gas resources beneath the oceans, prioritizing the efficient and convenient transportation of subsea oil and gas resources^3^. Compared to traditional oil and gas separation transportation technology, multiphase transportation simplifies the transportation process and reduces maintenance costs. Multiphase pumps, as the core equipment of multiphase transportation technology, have been optimized and improved significantly in recent years, not only enhancing the production efficiency but also significant economic benefits^4–6^. A tip leakage flow is generated in a multiphase pump due to the pressure difference and relative movement between the blade tip and the pump body wall of the impeller, and this interacts with the main flow to form the complex TLV^7^. The TLV destabilizes the flow and induces cavitation, vibration, and noise, all of which deteriorate the hydraulic mechanical performance^8–10^.

Many scholars have carried out extensive research on the multiphase pump, including gas–liquid two-phase flow^11^, bubble motion^12^, cavitation^13^ and energy conversion^14^. Zhang et al*.*^15–17^ noticed a visual change in the flow pattern of gas in a multiphase pump, with distinct isolated bubbles, bubbly flow, gas pockets, and segregated gas flow stages as the *IGVF* increased. They discovered that the bubble diameter is closely related to the *IGVF* and flow speed. Zhang et al*.*^18,19^ examined the bubble movement in the pump and reported that the bubbles moved and aggregated from the pressure surface (PS) to the suction surface (SS) of the blade, and the bubble volumes are significantly affected by the blade wall. Shi et al*.*^20^ found that under gas–liquid two-phase conditions, the impeller power degraded, and the energy conversion performance was worst. In the previous work^21^, the spatial–temporal evolution and dynamics of the TLV in multiphase pumps were revealed. However, the studies on the influence factors of the TLV are rarely reported, and there is a lack of systematic research on energy dissipation caused by the TLV.

Meticulous research survey indicated that the flow rate^22^, tip clearance^23^, and *IGVF*^13^ play a crucial role in affecting the flow pattern of the TLV and cause energy dissipation. Some studies^24–27^ have optimized the performance of pumps by quantitatively analyzing the position and mode of energy loss using entropy production theory. Ji et al*.*^28,29^ found that the high energy dissipation in the impeller of the mixed-flow pump is caused by TLV, secondary flow vortex, and stall vortex. As the tip clearance increases, the intensity and scale of the TLV surge, sharply increasing the total entropy generation. The dissipation of vortex energy is termed enstrophy, for which a theoretical formula was derived by Wu et al*.*^30^ derived from the kinetic energy transport equation, providing a theoretical basis for the study of turbulent enstrophy dissipation^31^. The relationship between enstrophy and energy dissipation rate in turbulent flow has been investigated, with enstrophy in the boundaries of objective Eulerian coherent structures produced by vortex stretching, and the enstrophy is transferred through the structure boundaries by viscous diffusion^32–34^. Lin et al*.*^35^ effectively diagnosed the flow characteristics of a turbine pump using enstrophy dissipation theory and determined the region of high enstrophy dissipation. Therefore, enstrophy dissipation theory is applied to the energy dissipation generated by the TLV, and the position, form, and dissipation rate can be determined.

In the present study, the enstrophy dissipation theory is innovatively applied to study the energy dissipation of the TLV quantitatively. The effects of the flow rate, tip clearance, and *IGVF* on the vorticity, flow pattern, pressure load, and enstrophy dissipation are analyzed in the impeller. A simple nonlinear regression model was established considering the influencing factors that accurately predict the enstrophy dissipation of the TLV. Finally, the enstrophy dissipation law under the interaction of multiple factors is further explored, and a multiple nonlinear regression model is established. The results presented provide a theoretical basis for calculating the energy dissipation of the TLV accurately in multiphase pumps.

## Methods

### Physical model and grid generation

**Figure 1:**
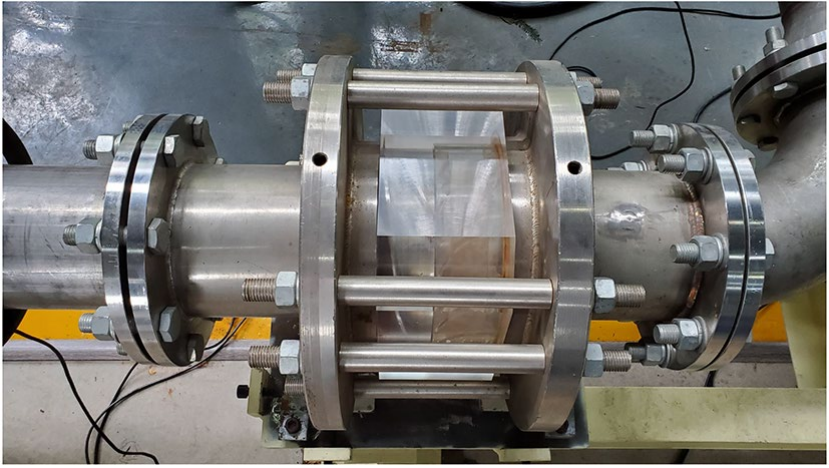
Test prototype of the multiphase pump.

**Figure 2:**
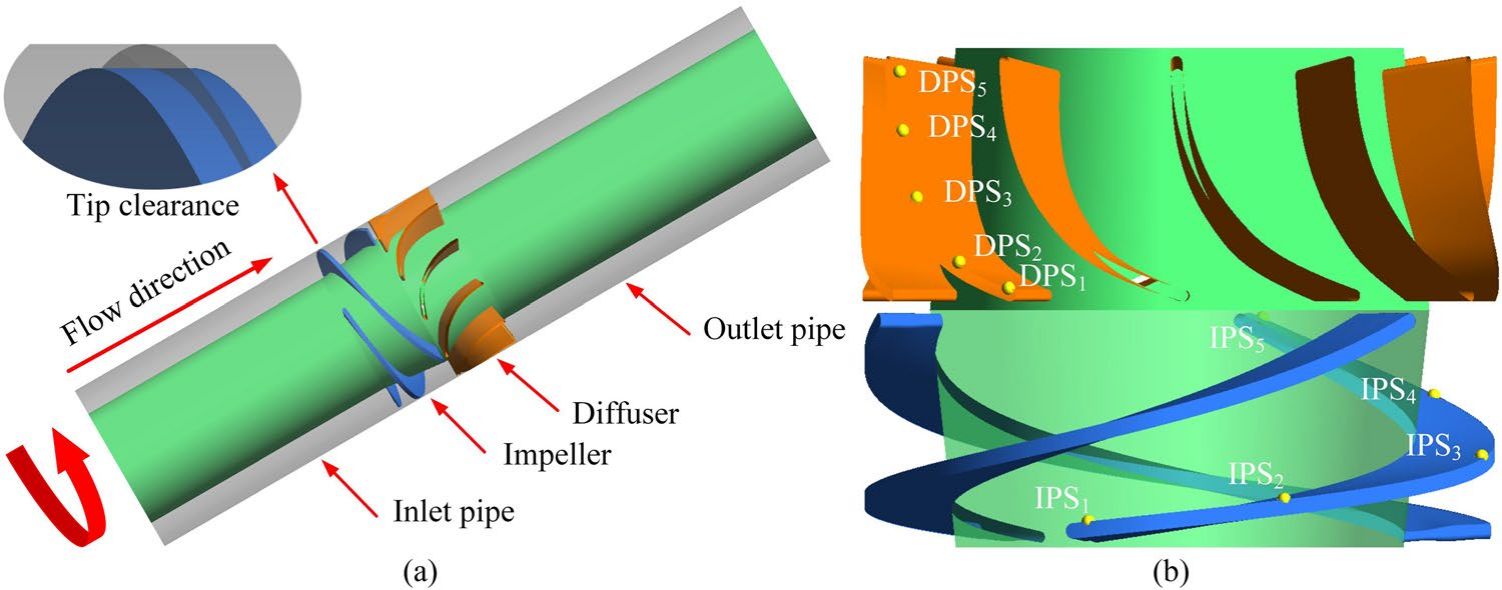
Schematic diagram of computational domain and monitoring points. (**a**) Computational domain and (**b**) monitoring points.

The research object is a self-designed single-stage multiphase pump, the main components of which include the impeller, diffuser, pump body, and inlet and outlet pipeline. The main design parameters are listed in Table 1, and the test prototype is shown in Fig. 1. A three-dimensional model for the computational domain, including the inlet, impeller, diffuser, and outlet domains, is developed using UG software (Fig. 2a). In addition, five pressure monitoring points are arranged on the PS of the impeller and diffuser, namely, IPS_1_–IPS_5_ and DPS_1_–DPS_5_, respectively, as shown in Fig. 2b.

**Table 1:** Main design parameters.

Parameter	Symbol	Value	Units
Design flow rate	*Q*_*d*_	100	m^3^∙h^−1^
Design speed	*n*	3600	rpm
Number of impeller blades	*Z*_1_	3	–
Number of diffuser blades	*Z*_2_	11	–
Hub/shroud ratio	*d̄*	0.7	–
Inner diameter	*D*	161	mm

**Figure 3:**
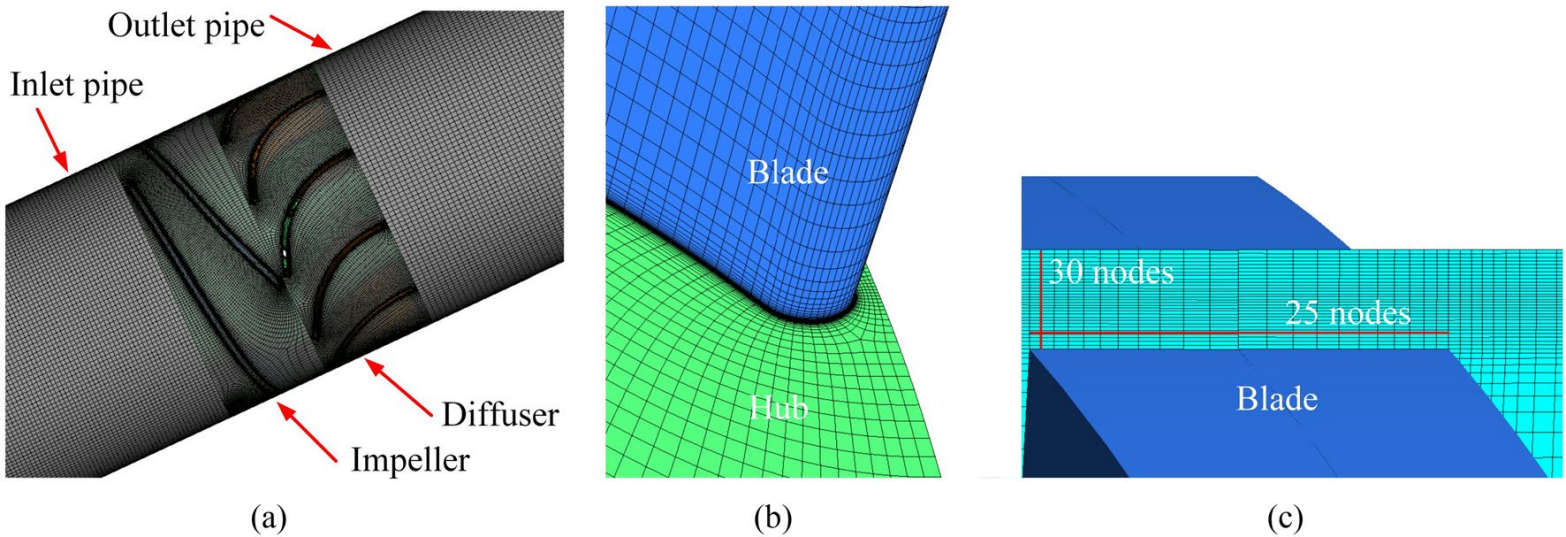
Computational grid. (**a**) Whole computational domain, (**b**) impeller blade, and (**c**) tip clearance.

A high-quality structural grid is critical for numerical simulations to be reliable and accurate. The grid covering the computational domain is arranged using a hexahedral structure, as shown in Fig. 3. The boundary layer grid near the wall is refined to ensure better quality, and the O-topology is used to surround the blade. In particular, 30 and 25 layers are arranged in the radial and circumferential directions of the tip clearance region, respectively. The value of the Y + is maintained at less than 60 in the impeller. The scalable wall function is used as a wall function, which is contributed to improving the computing accuracy.

### Numerical computation method

The numerical simulations of a multiphase pump were performed using ANSYS CFX. The Eulerian-Eulerian model is used to calculate gas–liquid two-phase flow^36^. The gas and liquid in the two-phase model were air and water, respectively, which are maintained at 25 °C. The ${\text{SST k}} - \omega$ turbulence model was used for the liquid phase, as it can effectively predict the flow separation point, separation region, and TLV trajectory. The gas-phase diameter was set to 0.1 mm, and the Dispersed Phase Zero Equation was used as its turbulence model. The rotational speed of the impeller was set to 3000 rpm in the pump, and the shroud of the impeller was set to counter-rotating mode in the relative coordinate system. Velocity inlet, pressure outlet, and no-slip wall conditions were imposed at the boundaries. The convergence precision was 10^−5^. Although smaller time steps capture unsteady information more accurately, they consume more computing resources. Thus, time steps of 5.56 × 10^−5^ s, 1.11 × 10^−4^ s, and 1.67 × 10^−4^ s corresponding to 360, 180, and 120 steps per revolution, respectively, were used to conduct simulations. The result shows the fluctuations in pressure at monitoring point DPS_1_ are almost similar under the different time steps, indicating that the influence of the time step size on the results is negligible. Ultimately, a time step of 1.11 × 10^−4^ s was selected for the transient simulations.

Five grid resolutions were selected for the simulations, and the results are presented in Table 2. As the grid resolution increases, the head and efficiency of the pump increase and tend to become stable. The difference in the head and efficiency between grids 4 and 5 is very small. The Grid Convergence Index (GCI) was used for quantifying the grid uncertainty^37–39^. The second, third, and fourth sets of grids were selected as the coarse grid, intermediate grid, and fine grid, respectively, and the total enstrophy dissipation power was selected as the extrapolated parameter. The analysis shows that the relative errors of the extrapolated parameter and the fine GCI are 0.114% and 0.466%, respectively. Therefore, given the computation time and accuracy requirements, the grid 4 was selected for the subsequent simulations.

**Table 2:** Grid independence study.

Component	Grid 1	Grid 2	Grid 3	Grid 4	Grid 5
Total number of cells	2,327,229	2,888,523	3,499,716	3,902,631	6,069,444
$P_{ens}$ (W)	1518.13	1580.21	1609.96	1628.45	1635.23
*η* (%)	43.35	44.49	44.60	45.41	45.58
$P_{ens}$/$P_{ens1}$	1	1.0409	1.0605	1.0727	1.0771
*η*/*η*_*1*_	1	1.0165	1.0218	1.0327	1.0384

**Figure 4:**
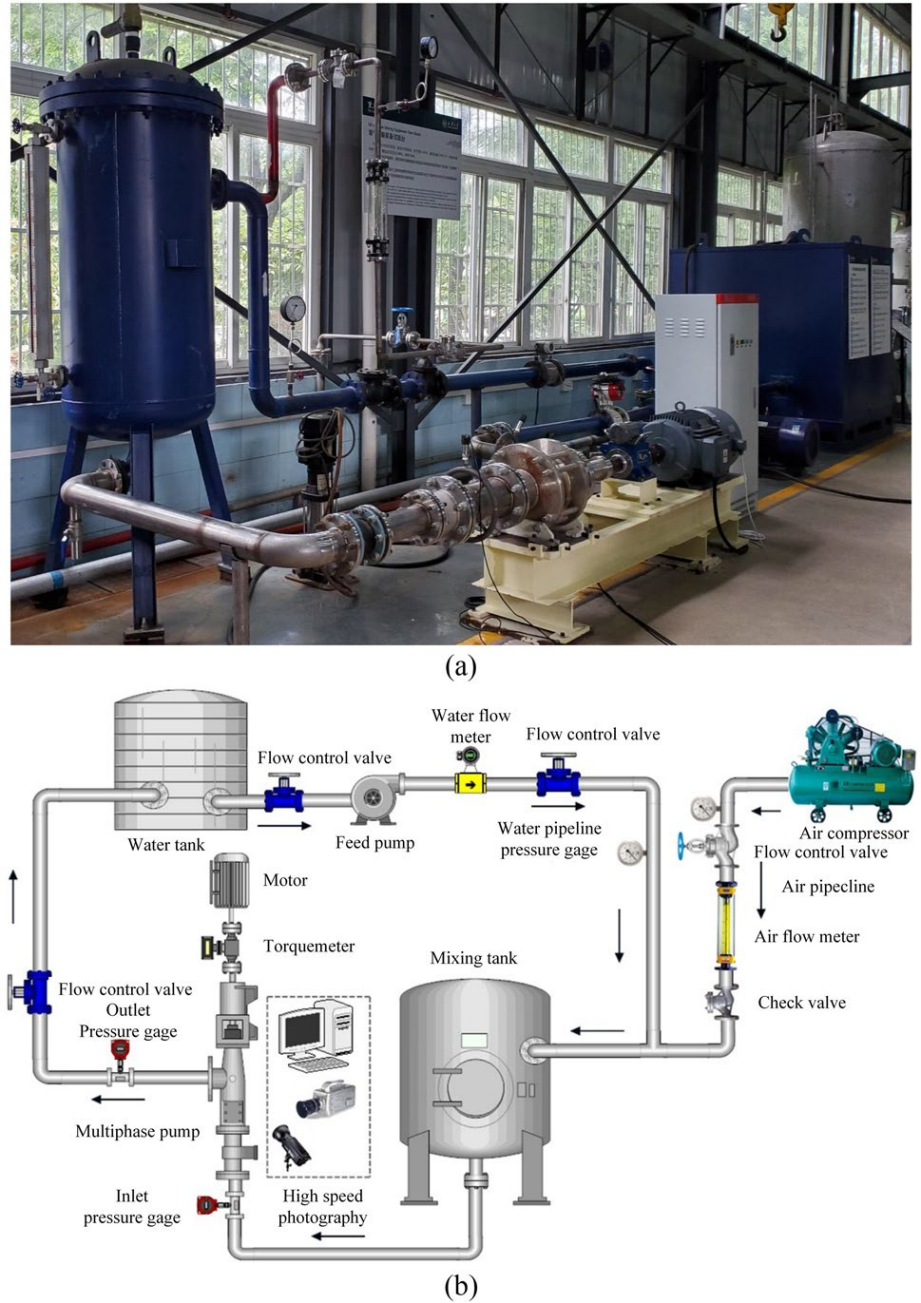
Test system of the multiphase equipment. (**a**) Photograph of the test system and (**b**) schematic of the test system.

Experimentation was conducted using a test system and multiphase equipment. As the system runs, air and water enter the mixing tank through the respective pipelines, and the valve is controlled to homogeneously mix the gas and liquid in different proportions. The test system is shown in Fig. 4, and the main performance indicators of the instruments are shown in Table 3. The calculation shows that the head uncertainty $\mu_{H}$ =  ± 0.98%, the power uncertainty $\mu_{P}$ =  ± 0.62%, and the efficiency uncertainty $\mu_{\eta }$ =  ± 1.25%.

**Table 3:** Main performance indicators of the instruments.

Instruments	Range	Precision	Unit
Inlet pressure gauge	0–0.8	0.3 class	MPa
Outlet pressure gauge	0–1	± 0.2%	MPa
Water flow meter	0–140	± 0.5%	m^3^/h
Airflow meter	0–60	1.5 class	m^3^/h
Torque meter	0–50	0.2 class	N∙m

**Figure 5:**
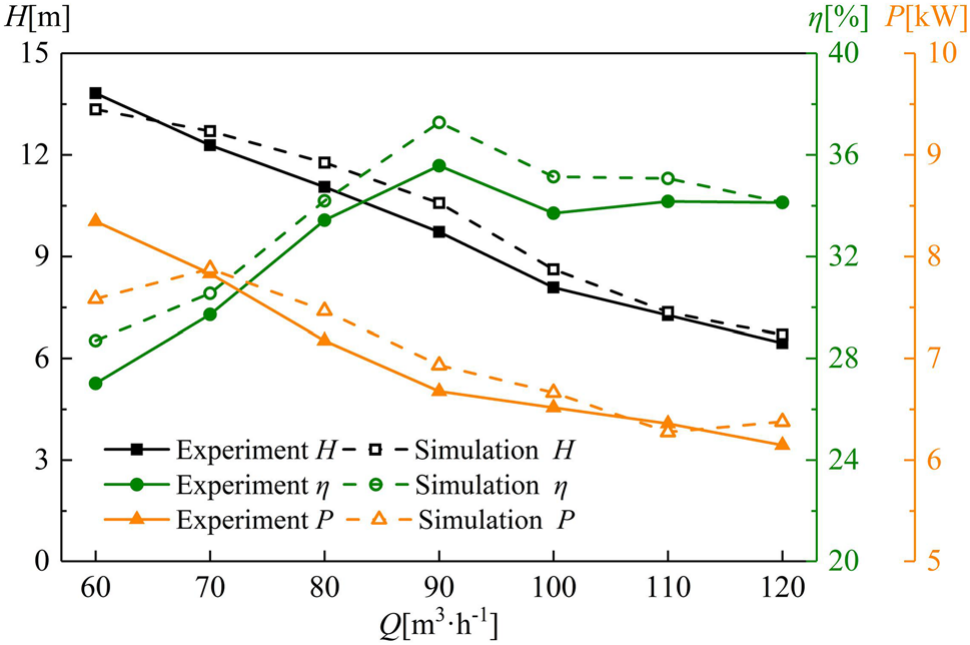
External characteristic lines of a multiphase pump.

The external characteristics of a multiphase pump with a tip clearance of 1.0 mm were tested, and values for the head, efficiency, and output power were obtained. The results of the test and simulation with external characteristic lines are shown in Fig. 5 for different flow rates of 60–120 m^3^h^−1^. The numerical simulation results are in good agreement with those of the experiments. Thus, it can be concluded that the numerical simulation scheme established in this study accurately predicts the energy performance of the multiphase pump.

### Ensrophy dissipation theory

To quantitatively analyze the influence of the tip leakage flow on the energy loss of a pump, the the kinetic energy transport equation was introduced:

1 $$ \rho \frac{DE}{{Dt}} = \rho f \cdot \mathop{u}\limits^{\rightharpoonup} + (\nabla \cdot \mathop{u}\limits^{\rightharpoonup} )p + \nabla \cdot (S_{T} \cdot \mathop{u}\limits^{\rightharpoonup} ) - \phi $$

In Eq. (1), $S_{T}$ is the stress tensor, given by $S_{T} = 2\mu S_{r} - (p - 2/3 \cdot (S_{T} \cdot \mathop{u}\limits^{\rightharpoonup} ))I$, where $\mu$, $S_{r}$, $p$, $\mathop{u}\limits^{\rightharpoonup}$, and $I$ denote the dynamic viscosity, strain tensor rate, pressure, circumferential velocity, and unit tensor, respectively. $\phi$ is the viscosity dissipation, expressed as $\phi = - 2\mu (\nabla \cdot \mathop{u}\limits^{\rightharpoonup} )^{2} /3 + 2\mu S_{r}$. If the compressibility of the fluid is ignored, $\phi$ is written as.

2 $$\phi \, = \,\mu \left| {\overset{\lower0.5em\hbox{$\smash{\scriptscriptstyle\rightharpoonup}$}} {\omega } } \right|^{2} \, - \,2\mu \nabla  \cdot ((\nabla  \cdot \overset{\lower0.5em\hbox{$\smash{\scriptscriptstyle\rightharpoonup}$}} {u} )I\, - \,\nabla \overset{\lower0.5em\hbox{$\smash{\scriptscriptstyle\rightharpoonup}$}} {u} ^{T} ) \cdot \overset{\lower0.5em\hbox{$\smash{\scriptscriptstyle\rightharpoonup}$}} {u}$$

where $\vec{\omega }$ is the vorticity. On substituting Eq. (2) in Eq. (1) and ignoring the effect of the body force, the kinetic energy equation of an incompressible fluid is obtained as follows:

3 $$ \rho \frac{DE}{{Dt}} = - \nabla \cdot (p\mathop{u}\limits^{\rightharpoonup} ) - \nabla \cdot (\mu \mathop{\omega }\limits^{\rightharpoonup}  \times \mathop{u}\limits^{\rightharpoonup} ) - \mu \left| {\mathop{\omega }\limits^{\rightharpoonup}  } \right|^{2} $$

In Eq. (3), $\nabla \cdot (p\mathop{u}\limits^{\rightharpoonup} )$ represents the work done by pressure on the fluid, called the *pressure propulsion work*. $\nabla \cdot (\mu \mathop{\omega }\limits^{\rightharpoonup}  \times \mathop{u}\limits^{\rightharpoonup} )$ denotes the nonlinear effects of vorticity and velocity on a viscous fluid. The third term, $\mu \left| {\mathop{\omega }\limits^{\rightharpoonup}  } \right|^{2}$, reflects the dissipation effect of the fluid viscosity and vorticity field on kinetic energy, which is called the *enstrophy dissipation rate*.

The volume enstrophy dissipation rate function is written as.

4 $$ \Phi_{k} = \mu \left[ {(\frac{\partial w}{{\partial y}} - \frac{\partial v}{{\partial z}})^{2} + (\frac{\partial u}{{\partial z}} - \frac{\partial w}{{\partial x}})^{2} + (\frac{\partial v}{{\partial x}} - \frac{\partial u}{{\partial y}})^{2} } \right] $$

where $u$, $v$, and $w$ represent the velocity components along the $x$, $y$, and $z$ directions in the Cartesian coordinate system, respectively.

For turbulent flow, the volume enstrophy dissipation rate can be divided into two parts:

5 $$ \Phi_{k} = \Phi_{ave} + \Phi_{ful} $$

where $\Phi_{ave}$ represents the average enstrophy dissipation rate and $\Phi_{ful}$ represents the fluctuating enstrophy dissipation rate, which can be given as follows:

6 $$ \Phi_{ave} = \mu \left[ {(\frac{{\partial \overline{w}}}{\partial y} - \frac{{\partial \overline{v}}}{\partial z})^{2} + (\frac{{\partial \overline{u}}}{\partial z} - \frac{{\partial \overline{w}}}{\partial x})^{2} + (\frac{{\partial \overline{v}}}{\partial x} - \frac{{\partial \overline{u}}}{\partial y})^{2} } \right] $$

7 $$ \Phi_{ful} = \mu \left[ {\overline{{(\frac{\partial w}{{\partial y}} - \frac{\partial v}{{\partial z}})^{2} }} + \overline{{(\frac{\partial u}{{\partial z}} - \frac{\partial w}{{\partial x}})^{2} }} + \overline{{(\frac{\partial v}{{\partial x}} - \frac{\partial u}{{\partial y}})^{2} }} } \right] $$

Kock and Herwig^40^ found that the rate of fluctuating enstrophy dissipation is closely related to the turbulence model used in numerical simulations. Therefore, the fluctuating enstrophy dissipation rate is defined as the product of the fluid density $\rho$ and turbulent dissipation rate $\varepsilon$:

8 $$ \Phi_{ful} = \rho \varepsilon $$

Energy dissipation on the walls cannot be ignored because of the no-slip conditions. Thus, the wall enstrophy dissipation rate proposed by Hou et al*.* was adopted:^24^.

9 $$ \Phi_{wall} = \tau \cdot v $$

Equations (6), (8), and (9) can be combined to obtain the average enstrophy dissipation power $P_{ave}$, fluctuating enstrophy dissipation power $P_{ful}$, and wall enstrophy dissipation power $P_{wall}$:

10 $$ P_{ave} = \int_{0}^{V} {\Phi_{ave} dV} $$

11 $$ P_{ful} = \int_{0}^{V} {\Phi_{ful} dV} $$

12 $$ P_{wall} = \int_{0}^{S} {\Phi_{wall} dS} $$

The volume enstrophy dissipation power $P_{k}$ and total enstrophy dissipation power $P_{ens}$ are given by:

13 $$ P_{k} = P_{ave} + P_{ful} $$

14 $$ P_{ens} = P_{ave} + P_{ful} + P_{wall} $$

## Discussions

### Influence of flow rate on enstrophy dissipation

**Figure 6:**
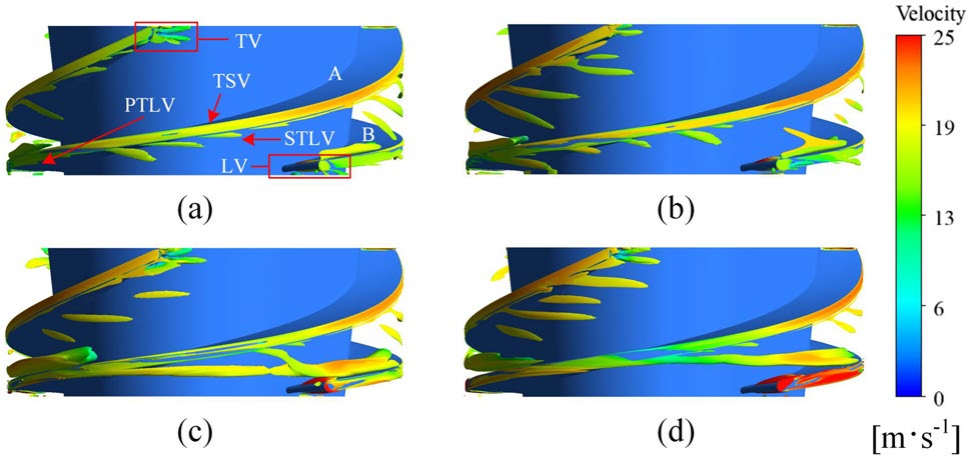
Flow pattern of the TLV. (**a**) 80 m^3^∙h^−1^, (**b**) 90 m^3^∙h^−1^, (**c**) 100 m^3^∙h^−1^, and (**d**) 110 m^3^∙h^−1^.

As illustrated in Fig. 6, the isosurface of the TLV is defined using *Q*_*c*_ criterion (*Q*_*c*_ = 1.5 × 10^6^ s^−2^), and the relative velocity is applied to color the TLV. As the flow rate increases, the leakage flow is enhanced by the impact of the main flow, the flow pattern becomes more disordered, the TLV scale increases, the inlet velocity of the impeller increases, finally enhancing the resistance effect on the PTLV.

**Figure 7:**
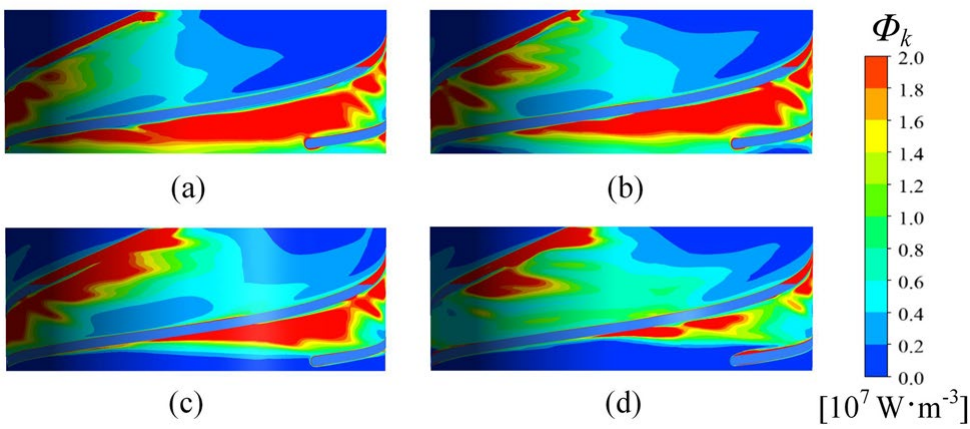
Distribution of the volume enstrophy dissipation rate at 0.95 span. (**a**) 80 m^3^∙h^−1^, (**b**) 90 m^3^∙h^−1^, (**c**) 100 m^3^∙h^−1^, and (**d**) 110 m^3^∙h^−1^.

The volume enstrophy dissipation rate $\Phi_{k}$ at 0.95 span is shown in Fig. 7. A larger pressure difference creates a stronger leakage flow intensity and greater enstrophy dissipation. Therefore, $\Phi_{k}$ is concentrated on the SS of the blade and coincides with the flow pattern of the TLV. An increase in the flow rate makes the distribution of $\Phi_{k}$ become more dispersed, and the area of high $\Phi_{k}$ gradually decreases, while the area with medium values of $\Phi_{k}$ gradually expands. The fundamental reason for the dispersive distribution of $\Phi_{k}$ is the presence of small-scale vortices in the impeller passage.

**Figure 8:**
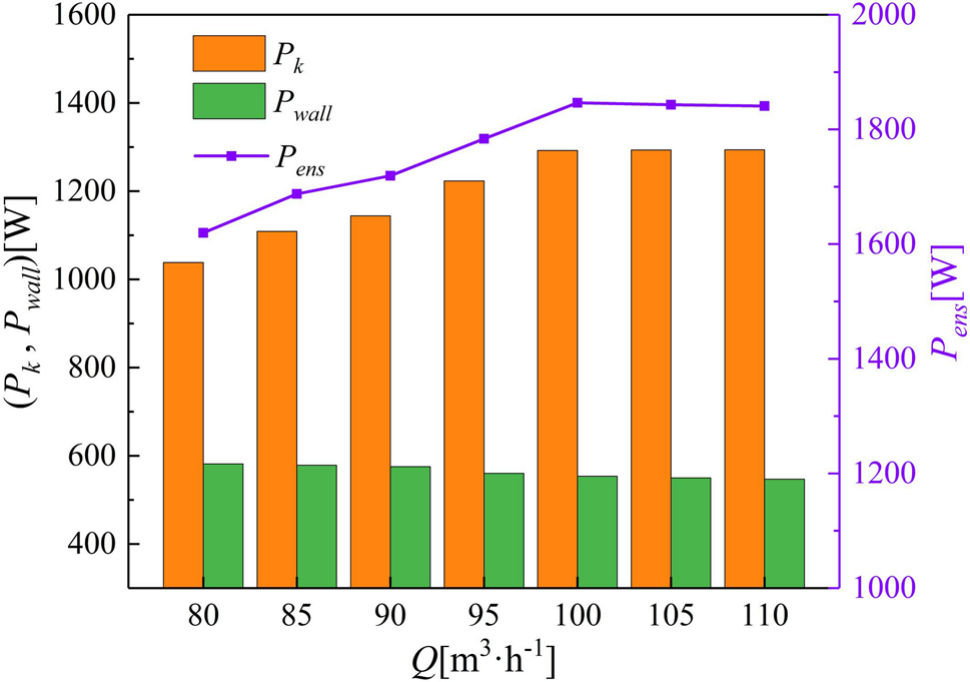
Variation in enstrophy dissipation power with flow rate.

Figure 8 shows a histogram of the volume enstrophy dissipation power $P_{k}$ and the wall enstrophy dissipation power $P_{wall}$, and the variation in the total enstrophy dissipation power $P_{ens}$. The leakage rate and gas phase are the key factors affecting $P_{k}$. The results show that $P_{k}$ increases with flow rate. However, the crucial factors affecting $P_{wall}$ are the shear stress, leakage rate, and action area. As the flow rate increases, both the shear stress and leakage flow increase. The pump body wall area typically shows the high $\Phi_{wall}$. In addition, $\Phi_{wall}$ is affected by the gas phase attached to the walls in the impeller, which effectively reduces the action area between the liquid phase and the walls, resulting in a reduction in $\Phi_{wall}$.

### Influence of tip clearance on enstrophy dissipation

**Figure 9:**
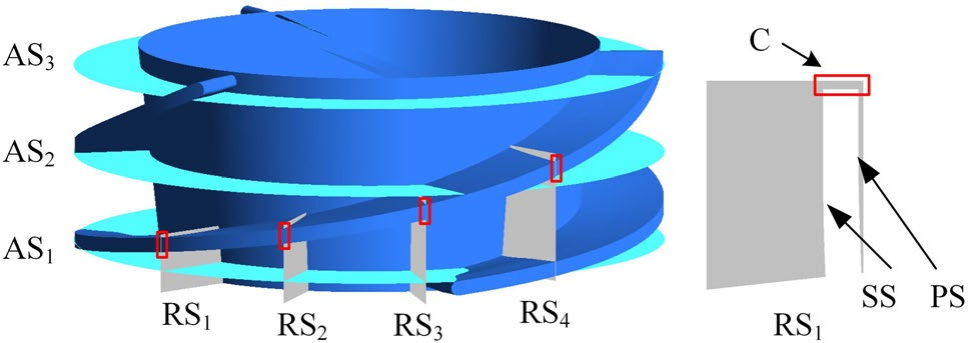
Schematic diagram of the sections in the impeller.

**Figure 10:**
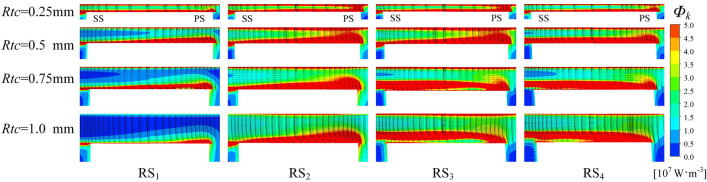
Volume enstrophy dissipation rate and velocity vector in section C.

A total of four radial sections, denoted as RS_1_–RS_4_ in turn, are evenly distributed in the trajectory area of the PTLV, with the axial distance of the sections spanning only the flow passage between two blades. Three axial sections are selected with axial lengths of 10%, 50%, and 90% denoted as AS_1_–AS_3_, respectively, in the impeller between the inlet and outlet (Fig. 9). The volume enstrophy dissipation rate $\Phi_{k}$ and velocity vector under different tip clearances are displayed in Fig. 10. The TSV forms in the tip clearance region and the vortex region are quite similar to the enstrophy dissipation region. Therefore, the TSV in the tip clearance region is primarily responsible for energy dissipation. From RS_1_ to RS_3_, the pressure difference of the tip clearance increases gradually, and the vorticity and scale of the TSV increase accordingly, resulting in a continuous increase in $\Phi_{k}$. As the tip clearance increases, the STLV scale increases and extends to the SS of the blade. This proves that the enstrophy dissipation increases as the tip clearance increases.

**Figure 11:**
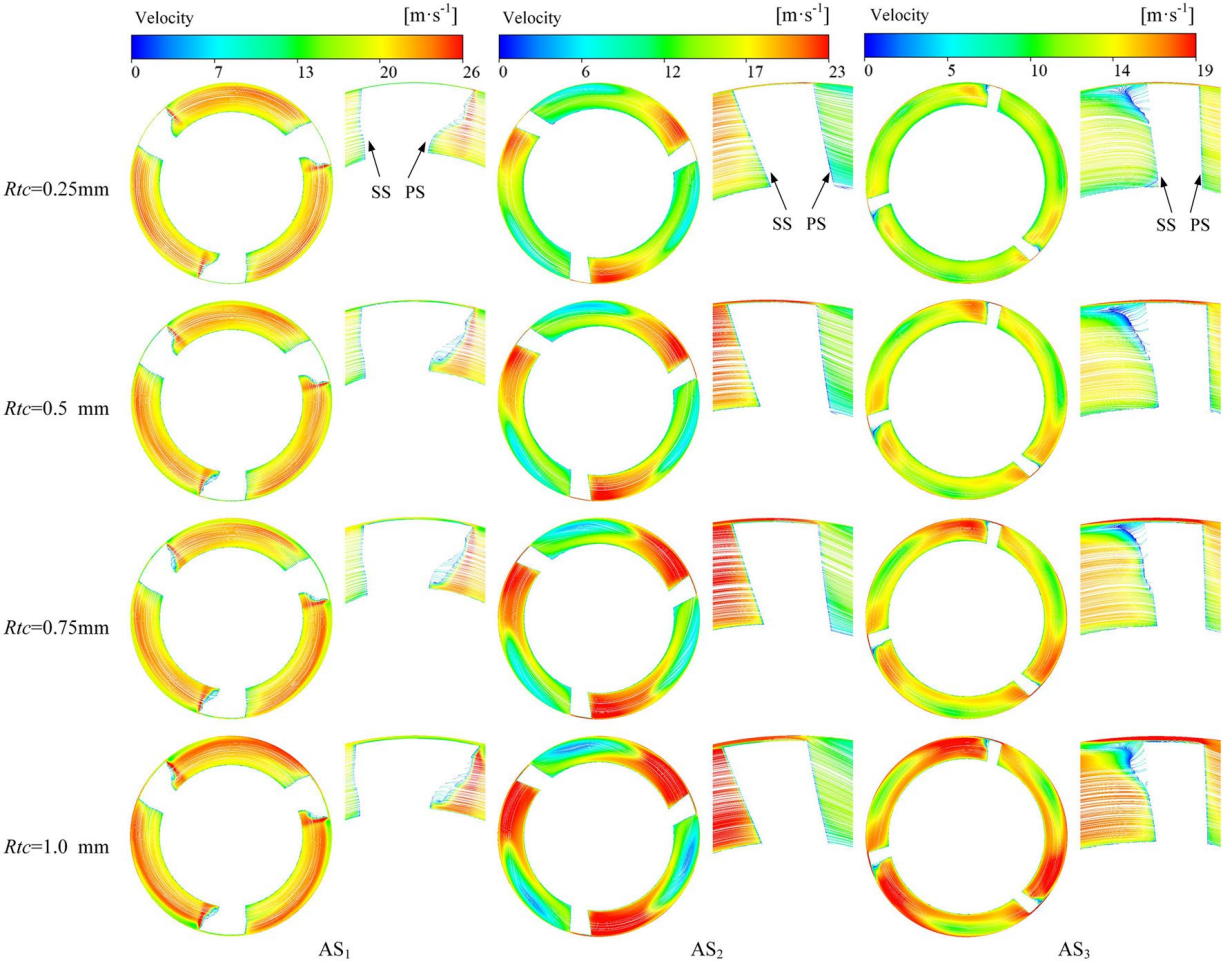
Velocity streamlines on axial sections.

The streamline distributions on AS_1_–AS_3_ are shown in Fig. 11. The velocity distributions on AS_1_–AS_3_ are closely related to the flow pattern of the TLV along the direction of the main flow. The velocity is uniform at the impeller inlet (AS_1_). With increasing tip clearance, the velocity in the middle of the impeller passage gradually increases, while the low-speed region shrinks in size. Simultaneously, an obvious gradient appears in the velocity distribution in the middle of the impeller (AS_2_). A high-speed region appears on the SS, while a large low-speed region is generated on the PS. The high-speed and low-speed region gradually expands with increasing tip clearance. Furthermore, an obvious vortex core with low velocity appears when the tip clearance is 1 mm, with increasing velocity in the radial direction centered on the vortex core. Finally, the velocity tends to be uniform at the impeller outlet (AS_3_), with high-speed regions appearing on both PS and SS. The velocity of the TV generated on the SS is the lowest.

**Figure 12:**
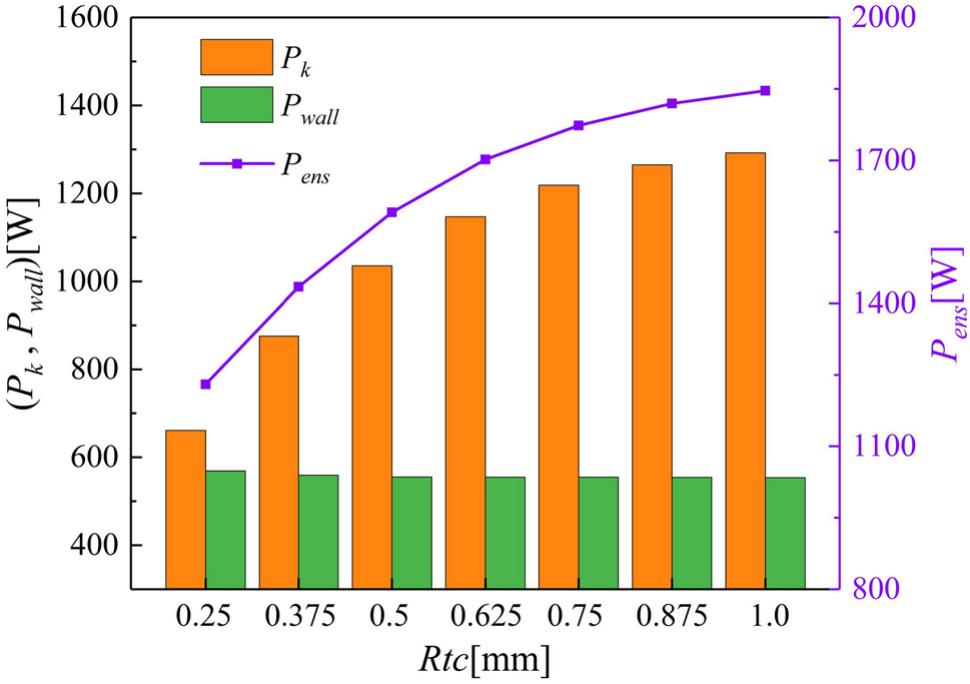
Variation in enstrophy dissipation power with tip clearance.

Figure 12 shows a histogram of the volume enstrophy dissipation power $P_{k}$, the wall enstrophy dissipation power $P_{wall}$, and variations in the total enstrophy dissipation power $P_{ens}$. Even minute variations in the tip clearance cause significant changes to the enstrophy dissipation in the impeller. As the tip clearance increases, the leakage rate increases, and the vorticity and scale of the TLV increase accordingly, thus, enhancing $P_{k}$. However, the volume increment decreases significantly, indicating that the sensitivity of $P_{k}$ to the tip clearance gradually decreases. Moreover, when the tip clearance is small, the jet effect near the pump body wall is stronger, allowing the generation of small-scale vortex groups and resulting in an increase in $P_{wall}$. Therefore, $P_{wall}$ decreases with increasing tip clearance.

### Influence of IGVF on enstrophy dissipation

To quantitatively analyze the correlation between the pressure fluctuation and enstrophy dissipation in the impeller of a multiphase pump, the pressure fluctuation intensity is introduced and given as follows:

15 $$ \overline{p} { = }\frac{1}{N}\sum\limits_{i = 1}^{n} {p_{i} } $$

16 $$ \overline{{p^{\prime}}} { = }\sqrt {\frac{1}{N}\sum\limits_{i = 1}^{n} {(p_{i} - \overline{p} )^{2} } } $$

where $N$ is the number of sample points in the statistical period, $p^{\prime}$ is the pressure value of each time step, and $\overline{p}$ is the arithmetic mean in the statistical period. The dimensionless pressure fluctuation intensity is given by.

17 $$ I_{PF} = \frac{{\overline{{p^{\prime}}} }}{{\frac{1}{2}\rho U_{tip}^{2} }} $$

where $U_{tip}^{{}}$ is the tip circumferential velocity at a tip clearance of 1.0 mm (24.96 m/s in this study) and $\rho$ is the density of the liquid.

**Figure 13:**
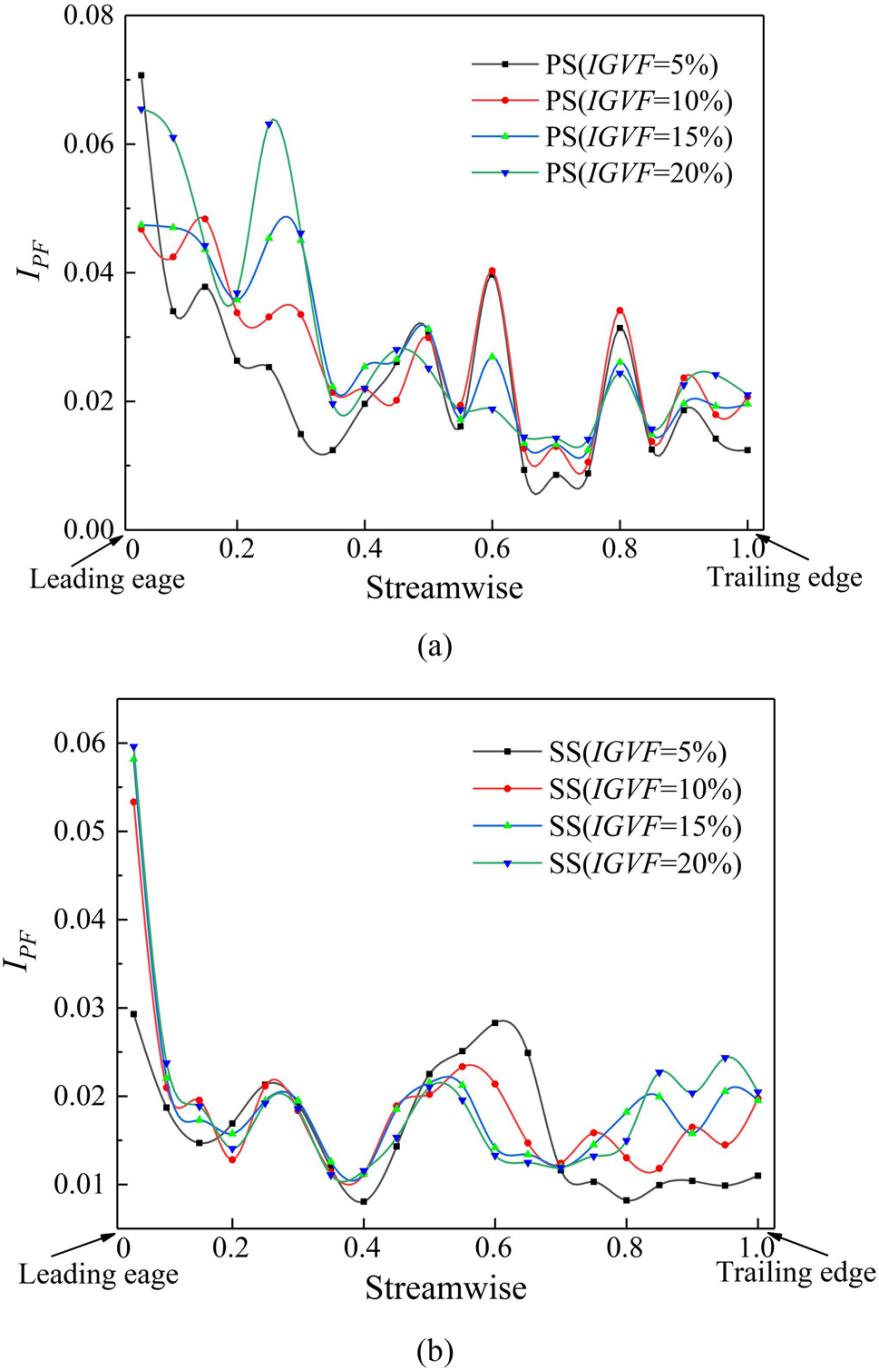
Pressure fluctuation intensity on the blade. (**a**) Pressure fluctuation intensity on the PS and (**b**) Pressure fluctuation intensity on the SS.

Twenty monitoring points were evenly distributed at the tip between the leading and trailing edges of the PS and SS, and the angle between each two monitoring points is 10º. the curves of pressure fluctuation intensity $I_{PF}$ under varied *IGVF* conditions were recorded (Fig. 13). It is apparent that the LV generated by the attack angle between the main flow direction and the setting angle of the blade deteriorates the flow pattern in the tip region, resulting in high $I_{PF}$ values at the leading edge. Moreover, the flow state gradually becomes more stable from the leading edge to the trailing edge, while the TSV generated by the tip clearance disturbs the flow field, causing oscillations in $I_{PF}$ on the PS. The increased $I_{PF}$ in the PS with IGVF reflects the increase in the gas phase, enhancing the vorticity and scale of the TLV, which disorders the flow in the tip region and increases enstrophy dissipation.

**Figure 14:**
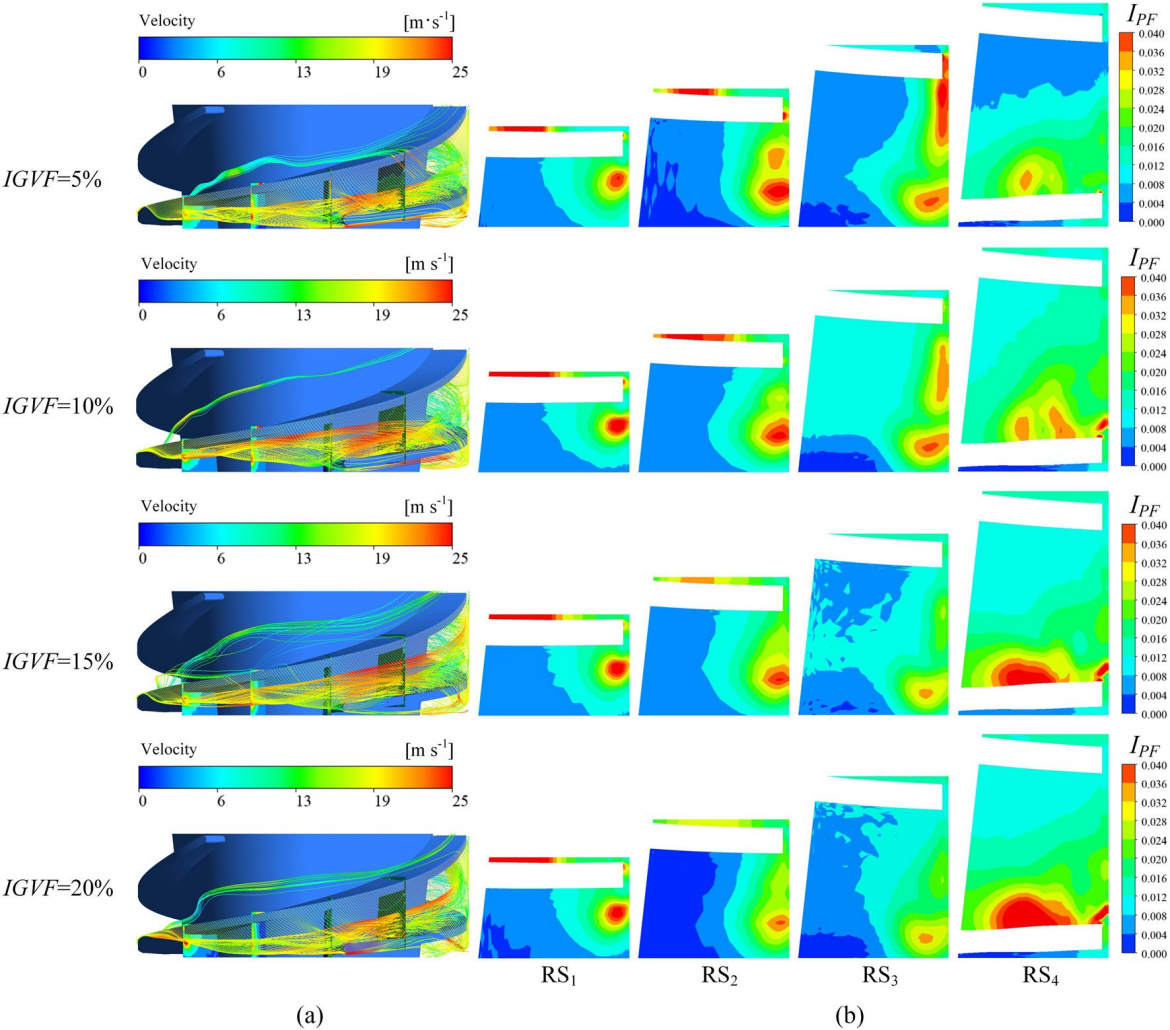
Three-dimensional streamline in the impeller and pressure fluctuation intensity on radial sections. (**a**) Three-dimensional streamline and (**b**) pressure fluctuation intensity.

A line segment extending from the leading edge to the trailing edge in the tip clearance region is generated and the three-dimensional streamlines released from this line segment along with the pressure fluctuation intensity $I_{PF}$ on RS_1_–RS_4_ are shown in Fig. 14. This figure visualizes the process of TLV formation by the interaction between the tip leakage flow and the main flow. The PTLV originates at the leading edge and moves downstream along the direction of the main flow with a gradual decrease in its vorticity, whereas its vortex scale increases continuously. As the *IGVF* increases, the initial point of the PTLV gradually moves to the trailing edge, and its flow pattern becomes highly disordered, leading to enhanced enstrophy dissipation. When the patterns on RS_1_–RS_4_ are compared, it is found that $I_{PF}$ is highest at the TLV core and gradually weakens along the flow direction, indicating that $I_{PF}$ in the TLV core is closely related to vorticity.

**Figure 15:**
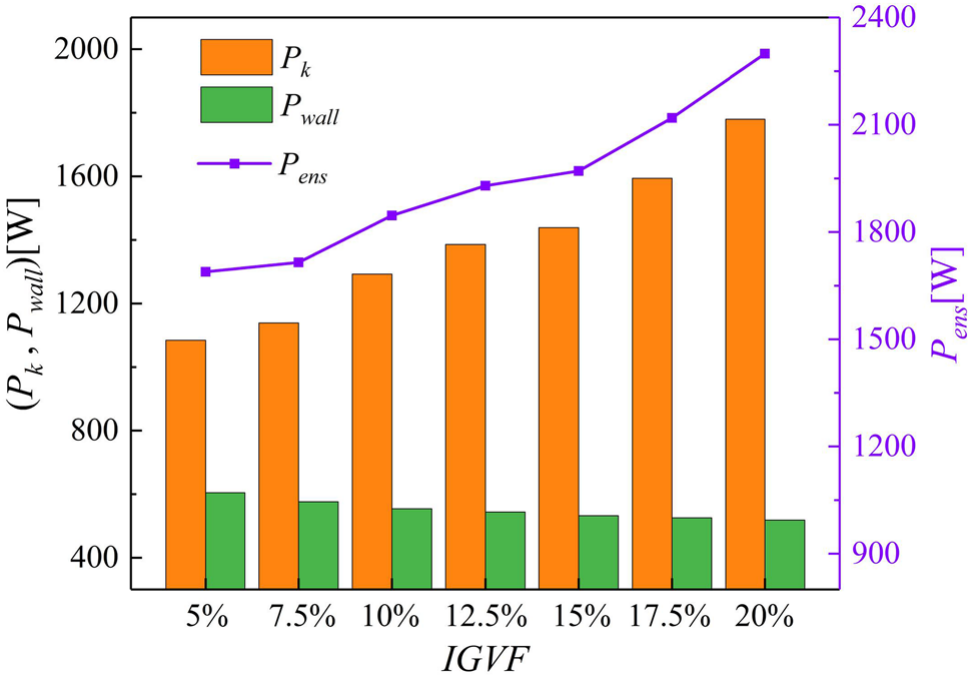
Variation in enstrophy dissipation power with *IGVF.*

Figure 15 shows a histogram of the volume enstrophy dissipation power $P_{k}$, the wall enstrophy dissipation power $P_{wall}$, and the variations in the total enstrophy dissipation power $P_{ens}$. The increment in $P_{k}$ is smaller under lower *IGVF* conditions (5–15%), with a significant increase shown upon reaching 20% *IGVF*. This indicates that the performance of the multiphase pump decreases significantly under high *IGVF* conditions*.* Most importantly, the variation of $P_{ens}$ grows exponentially, indicating that higher *IGVF* conditions inevitably lead to greater energy loss. Therefore, the *IGVF* is the key factor affecting the power performance of the impeller.

### Simple nonlinear regression model

The flow rate, tip clearance, and *IGVF* are important factors affecting enstrophy dissipation in the impeller. Therefore, these three factors are selected to establish a simple nonlinear regression model to predict the variation law of enstrophy dissipation. Simple regression models for $P_{k}$, $P_{wall}$, and $P_{ens}$ were obtained. These models are given in Table 4.

**Table 4:** Simple regression models.

Variable	Model	Expression
*Q*	*P*_*k*_	− 82,204.44/*Q* + 2074.21
	*P*_*wall*_	− 1.3*Q* + 687.61
	*P*_*ens*_	− 70,735.42/*Q* + 2515.92
*Rtc*	*P*_*k*_	844.27*Rtc*^3^ − 2790.32*Rtc*^2^ + 3221.9*Rtc* + 16.41
	*P*_*wall*_	4.91/*Rtc* + 547.64
	*P*_*ens*_	708.12*Rtc*^3 ^− 2485.55*Rtc*^2^ + 299.63*Rtc* + 623.74
*IGVF*	*P*_*k*_	913.55*e*^3.24*IGVF*^
	*P*_*wall*_	432.5*IGVF*^*−*0.11^
	*P*_*ens*_	1497.98*e*^2.02*IGVF*^

These regression models were subjected to statistical tests to determine their predictive power with the given sample data. The results are presented in Table 5. The goodness of fit of all regression models is greater than 0.9, indicating that they can explain more than 90% of the variation of variables. The variance significance of the regression models is less than 0.05, which proves that the functional relationships between the variables and the models are significant. In addition, the residuals of the regression models are small and conform to a normal distribution.

**Table 5:** Statistical tests of regression models.

Variable	Model	*R*^*2*^	*F*	Sig	SSE
*Q*	*P*_*k*_	0.949	92.60	0	3289.08
	*P*_*wall*_	0.949	93.62	0	63.50
	*P*_*ens*_	0.940	78.51	0	2872.46
$Rtc$	*P*_*k*_	1.000	–	0	0
	*P*_*wall*_	0.924	60.84	0.01	13.43
	*P*_*ens*_	1.000	–	0	0
$IGVF$	*P*_*k*_	0.985	333.38	0	5492.29
	*P*_*wall*_	0.995	1057.42	0	25.80
	*P*_*ens*_	0.971	168.48	0	8453.41

**Figure 16:**
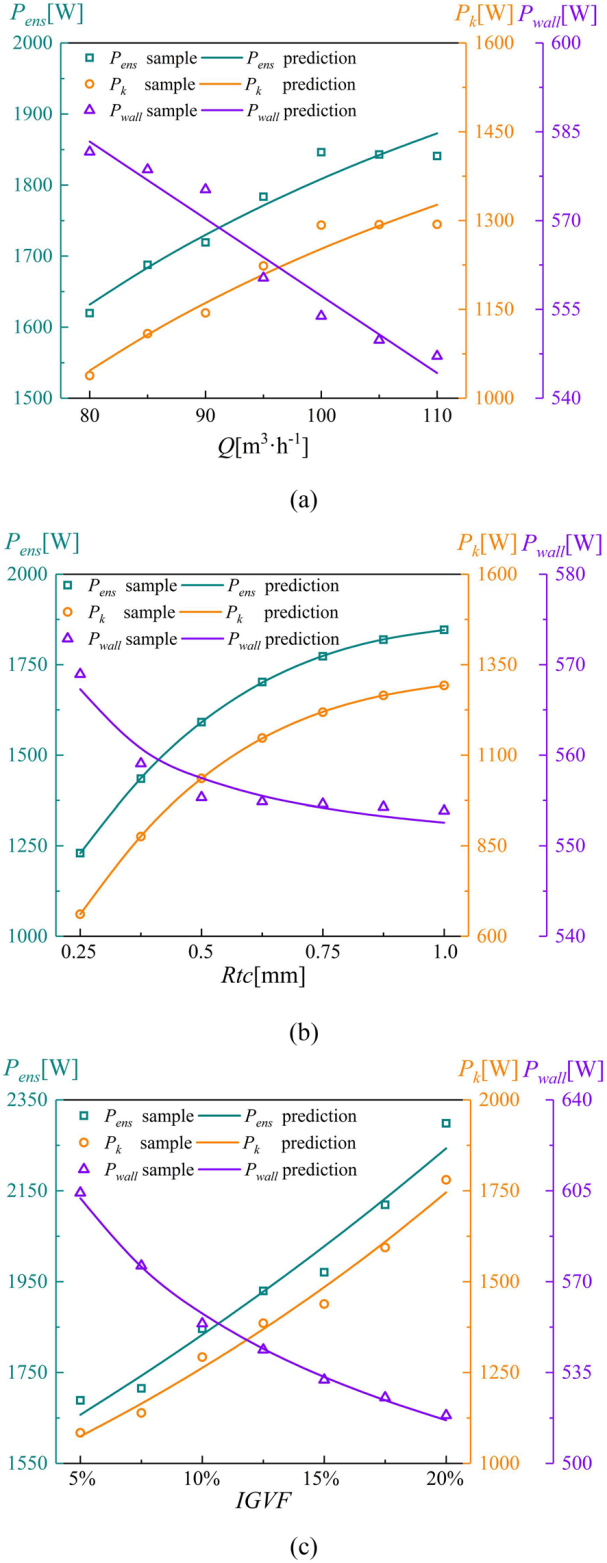
Prediction curves of the enstrophy dissipation power with different variables. (**a**) Flow rate, (**b**) tip clearance, and (**c**) *IGVF.*

The predicted enstrophy dissipation power under different variables is shown in Fig. 16. Figure 16a shows that there is an inverse relationship between $P_{k}$ and $Q$. Besides, there is a negative linear correlation between $P_{wall}$ and $Q$. As shown in Fig. 16b, both $P_{k}$ and $P_{ens}$ have a cubic relationship with *Rtc*, whereas $P_{wall}$ and *Rtc* are inversely related. Both $P_{k}$ and $P_{ens}$ gradually increase at a decreasing rate as *Rtc* increases, whereas $P_{wall}$ converges to a definite value. Similarly, Fig. 16c shows that both $P_{k}$ and $P_{ens}$ are exponential functions of *IGVF*, whereas there is a power function relationship between $P_{wall}$ and *IGVF*. The average error of $P_{k}$, $P_{wall}$, and $P_{ens}$ is less than 2%. The result is indicated that the regression models have high prediction accuracy.

### Multiple nonlinear regression model

The flow rate, tip clearance, and *IGVF* change simultaneously and affect one another. Therefore, 16 conditions were selected to obtain sample values of the enstrophy dissipation power in the impeller. The results are listed in Table 5. Using the data in Table 6, the independent variables are first linearized. Then, a multiple linear regression method is used to fit the linearized variables. Finally, multiple nonlinear regression models are established for $P_{k}$, $P_{wall}$, and $P_{ens}$. The results are presented in Table 7.

**Table 6:** Influence of multiple variables on enstrophy dissipation power.

Number	*Q*[m^3^∙h^−1^]	*Rtc *[mm]	*IGVF*	*P*_*k*_ [W]	*P*_*wall*_ [W]	*P*_*ens*_ [W]
1	80	0.25	5%	629.30	623.49	1252.79
2	80	0.5	10%	979.24	585.42	1564.66
3	80	0.75	15%	1284.99	562.57	1847.56
4	80	1.0	20%	1700.24	539.93	2240.17
5	90	0.25	10%	655.12	581.58	1236.70
6	90	0.5	15%	1186.31	551.28	1737.59
7	90	0.75	20%	1565.89	531.08	2096.97
8	90	1.0	5%	1042.88	606.44	1649.32
9	100	0.25	15%	1104.61	555.85	1660.46
10	100	0.5	20%	1507.24	523.40	2030.64
11	100	0.75	5%	990.60	595.88	1586.48
12	100	1.0	10%	1292.44	553.86	1846.30
13	110	0.25	20%	1676.91	524.68	2201.59
14	110	0.5	5%	811.06	571.40	1382.46
15	110	0.75	10%	1161.59	553.14	1714.73
16	110	1.0	15%	1579.37	529.23	2108.60

**Table 7:** Multiple regression models.

Model	Expression
*P*_*k*_	5.88*Q* + 516.62*Rtc* + 285.34*e*^7*IGVF *^*− *521.07
*P*_*wall*_	− 1.1*Q* + 4.5/*Rtc* + 454.72*IGVF* + 713.85
*P*_*ens*_	4.78*Q* + 500.89*Rtc* + 197.88*e*^7*IGVF*^ + 399.99

**Figure 17:**
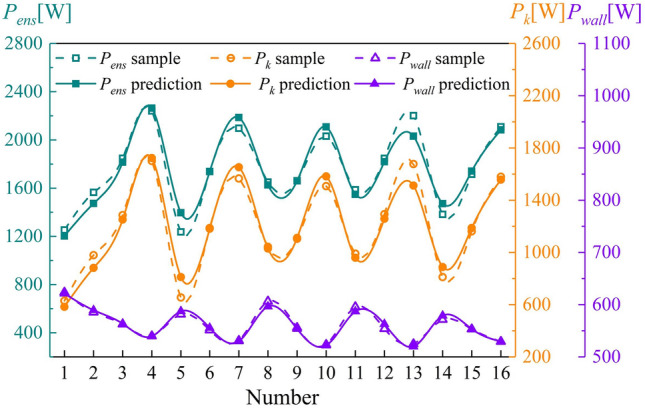
Comparison of sample values and predicted values given by multiple regression models.

The results of statistical tests performed on these regression models are presented in Tables 8 and 9. Table 8 shows that the adjusted *R*^*2*^ values of $P_{k}$, $P_{wall}$, and $P_{ens}$ exceed 0.9, indicating better goodness of fit. Moreover, the $F$ statistics of the regression models are high, and the correlation between the explained and explanatory variables is significant. The Durbin–Watson (DW) values of $P_{k}$ and $P_{ens}$ are close to 2, indicating that the residual sequence has no autocorrelation. Table 9 shows that the significance of the *T* statistics between variables is less than 0.05, suggesting the significant effect of the regression model. The tolerance and VIF values of all variables are 1 for $P_{k}$, $P_{wall}$, and $P_{ens}$, inferring that there is no collinearity between variables. The sample values are compared with the predicted values for multiple regression models in Fig. 17. The average error of $P_{k}$, $P_{wall}$, and $P_{ens}$ is less than 5%, indicating that the multiple regression models achieve high predictive accuracy.

**Table 8:** Significance test results.

Model	Adjusted *R*^*2*^	*F*	Sig	DW
*P*_*k*_	0.938	76.64	0	1.93
*P*_*wall*_	0.961	125.45	0	2.44
*P*_*ens*_	0.923	61.35	0	2.02

**Table 9:** Correlation test between variables.

Model	Variable	Coefficient	*T*	Sig	Tol	VIF
*P*_*k*_	*Q*	5.88	3.07	0.01	1	1
	*Rtc*	516.62	6.76	0	1	1
	*IGVF*	285.34	13.22	0	1	1
*P*_*wall*_	*Q*	− 1.10	− 8.31	0	1	1
	*Rtc*	4.50	3.54	0.004	1	1
	*IGVF*	− 454.72	− 17.17	0	1	1
*P*_*ens*_	*Q*	4.78	2.43	0.032	1	1
	*Rtc*	500.89	6.37	0	1	1
	*IGVF*	197.88	11.73	0	1	1

## Conclusions

In the present work, the enstrophy dissipation theory is innovatively applied to quantitatively study the energy dissipation of the TLV. The flow rate, tip clearance, and *IGVF* play a crucial role in impacting the flow pattern and enstrophy dissipation of the TLV. Simple and multiple nonlinear regression models for enstrophy dissipation are established considering the flow rate, tip clearance, and *IGVF* as independent variables, to accurately predict the enstrophy dissipation law. The results of the numerical simulation and experiment measurement are compared to verify the reliability and accuracy of the numerical simulation. Some important conclusions can be drawn as follows:

1. The initial point of the PTLV gradually moves from the leading edge to the trailing edge of the blade as the flow rate increases, decreasing the separation angle between the PTLV trajectory and the blade. It is observed that the distribution of the gas phase near the shroud is associated with the TLV trajectory. Gas void fraction is highest at the core of the TLV and gradually decreases along the radial direction with the vortex core as the center. Specifically, compared with the flow rate of 80 m^3^∙h^−1^, the volume enstrophy dissipation power increases by 24.63% with the flow rate of 110 m^3^∙h^−1^, and the wall enstrophy dissipation power decreases by 5.93%. On the whole, the total enstrophy dissipation power increases by 13.65%.
2. The jet effect on the walls is significant when the clearance is small, leading to the formation of small-scale vortices with counter-rotating directions in these regions boosting the enstrophy dissipation. As the tip clearance increases, the TSV scale increases and extends to the SS, and the velocity gradient in the impeller passage increases. Moreover, compared with the tip clearance of 0.25 mm, the volume enstrophy dissipation power increases by 95.52% with the tip clearance of 1 mm, and the wall enstrophy dissipation power decreases by 2.65%. On the whole, the total enstrophy dissipation power increases by 50.11%.
3. As the *IGVF* increases, the vorticity and scale of the STLV and pressure fluctuation intensity increase, disorganizing the flow pattern in the tip region. In particular, the STLV develops from a continuous sheet vortex to a scattered strip vortex, significantly increasing the volume enstrophy dissipation power. However, the pressure fluctuation intensity gradually decreases on the hub and shroud and reduces the wall enstrophy dissipation power. Additionally, compared with an IGVF of 5%, the volume enstrophy dissipation power increases by 64.15% with an IGVF of 20%, and the wall enstrophy dissipation power decreases by 14.18%. On the whole, the total enstrophy dissipation power increases by 36.12%.
4. Moreover, statistical tests show that the goodness of fit of all regression models is greater than 0.9, the variance significance of the regression models is less than 0.05, and the average error is less than 5%. Hence, simple and multiple nonlinear regression models have the ability to predict the enstrophy dissipation of the TLV accurately, which can form the basis for practical engineering applications.

## Data Availability

The datasets used and/or analysed during the current study available from the corresponding author on reasonable request.

## List of symbols

$\mu$: Dynamic viscosity [Pa∙s]
$S_{r}$: Strain rate tensor [—]
$$p$$: Pressure [Pa]
$$\mathop{u}\limits^{\rightharpoonup} $$: Circumferential velocity [m∙s^−^^1^]
$$\mathop{\omega }\limits^{\rightharpoonup} $$: Vorticity [s^−^^1^]
$\Phi_{k}$: Volumetric enstrophy dissipation rate [W∙m^−^^3^]
$\Phi_{ave}$: Average enstrophy dissipation rate [W∙m^−^^3^]
$\Phi_{ful}$: Fluctuating enstrophy dissipation rate [W∙m^−^^3^]
$\Phi_{wall}$: Wall enstrophy dissipation rate [W∙m^−^^2^]
$P_{ens}$: Total enstrophy dissipation power [W]
$P_{ave}$: Average enstrophy dissipation power [W]
$P_{ful}$: Fluctuating enstrophy dissipation power [W]
$P_{k}$: Volumetric enstrophy dissipation power [W]
$P_{wall}$: Wall enstrophy dissipation power [W]
$I_{PF}$: Pressure fluctuation intensity [—]
$$\rho$$: Density [kg∙m^−^^3^]
*Q*: Flow rate [m^3^∙h^−^^1^]
*Rtc*: Tip clearance [mm]
$$\tau$$: Shear stress [N]
$$v$$: Velocity [m∙s^−^^1^]
*Q*_*c*_: Vortex intensity [s^−^^2^]
TLV: Tip leakage vortex
PTLV: Primary tip leakage vortex
STLV: Secondary tip leakage vortex
TSV: Tip separated vortex
LV: Leading-edge vortex
TV: Trailing-edge vortex
IGVF: Inlet gas void fraction
PS: Pressure surface
SS: Suction surface

## References

[1] Suh J, Kim J, Choi Y, Joo W, Lee K (2017). A study on numerical optimization and performance verification of multiphase pump for offshore plant. Proc. Inst. Mech. Eng. Part A.

[2] Liu M, Tan L, Xu Y, Cao S (2020). Optimization design method of multi-stage multiphase pump based on Oseen vortex. J. Petrol. Sci. Eng..

[3] Xiao W, Tan L (2021). Design method of controllable velocity moment and optimization of pressure fluctuation suppression for a multiphase pump. Ocean Eng..

[4] Liu M, Tan L, Cao S (2020). Influence of viscosity on energy performance and flow field of a multiphase pump. Renew. Energ..

[5] Zhang J, Cai S, Li Y, Li Y, Zhang Y (2017). Optimization design of multiphase pump impeller based on combined genetic algorithm and boundary vortex flux diagnosis. J. Hydrodyn..

[6] Zhang J, Zhu H, Yang C, Li Y, Wei H (2011). Multi-objective shape optimization of helico-axial multiphase pump impeller based on NSGA-II and ANN. Energ. Convers. Manage..

[7] Shi G, Liu Z, Xiao Y, Li H, Liu X (2020). Tip leakage vortex trajectory and dynamics in a multiphase pump at off-design condition. Renew. Energ..

[8] Lei T, Zhifeng X, Yabin L, Yue H, Yun X (2018). Influence of T-shape tip clearance on performance of a mixed-flow pump. Proc. Inst. Mech. Eng. A.

[9] Guo Q, Zhou L, Wang Z (2016). Numerical evaluation of the clearance geometries effect on the flow field and performance of a hydrofoil. Renew. Energ..

[10] Ji L, Li W, Shi W (2020). Influence of tip leakage flow and inlet distortion flow on a mixed-flow pump with different tip clearances within the stall condition. Proc. Inst. Mech. Eng. Part A.

[11] Liu M, Tan L, Cao S (2019). Dynamic mode decomposition of gas-liquid flow in a rotodynamic multiphase pump. Renew. Energ..

[12] Wang Q, Yao W (2016). Computation and validation of the interphase force models for bubbly flow. Int. J. Heat Mass Tran..

[13] Shi G (2020). Effect of the inlet gas void fraction on the tip leakage vortex in a multiphase pump. Renew. Energ..

[14] Zhang J, Fan H, Zhang W, Xie Z (2019). Energy performance and flow characteristics of a multiphase pump with different tip clearance sizes. Adv. Mech. Eng..

[15] Zhang J, Cai S, Li Y, Zhu H, Zhang Y (2016). Visualization study of gas–liquid two-phase flow patterns inside a three-stage rotodynamic multiphase pump. Exp. Therm. Fluid Sci..

[16] Zhang J, Cai S, Zhu H, Zhang Y (2015). Experimental investigation of the flow at the entrance of a rotodynamic multiphase pump by visualization. J. Petrol. Sci. Eng..

[17] Shi Y, Zhu H, Zhang J, Zhang J, Zhao J (2018). Experiment and numerical study of a new generation three-stage multiphase pump. J. Petrol. Sci. Eng..

[18] Zhang W, Zhu B, Yu Z (2020). Characteristics of bubble motion and distribution in a multiphase rotodynamic pump. J. Petrol. Sci. Eng..

[19] Zhang W, Xie X, Zhu B, Ma Z (2021). Analysis of phase interaction and gas holdup in a multistage multiphase rotodynamic pump based on a modified Euler two-fluid model. Renew. Energ..

[20] Shi G (2020). Energy conversion characteristics of multiphase pump impeller analyzed based on blade load spectra. Renew. Energ..

[21] Shu Z, Shi G, Tao S, Tang W, Li C (2021). Three-dimensional spatial-temporal evolution and dynamics of the tip leakage vortex in an oil–gas multiphase pump. Phys. Fluids..

[22] Liu Y, Tan L (2019). Spatial-temporal evolution of tip leakage vortex in a mixed-flow pump with tip clearance. J. Fluid. Eng..

[23] Xu M, Cheng H, Ji B, Peng X (2020). LES of tip-leakage cavitating flow with special emphasis on different tip clearance sizes by a new Euler-Lagrangian cavitation model. Ocean Eng..

[24] Hou H (2016). Numerical analysis of entropy production on a LNG cryogenic submerged pump. J. Nat. Gas Sci. Eng..

[25] Huang J (2021). Design and experimental verification of variable-structure vortex tubes for valveless piezoelectric pump translating high-viscosity liquid based on the entropy generation. Sensors Actuat. A-Phys..

[26] Li D (2017). Entropy production analysis of hysteresis characteristic of a pump-turbine model. Energ. Convers. Manage..

[27] Wang C, Zhang Y, Zhang J, Zhu J (2020). Flow pattern recognition inside a rotodynamic multiphase pump via developed entropy production diagnostic model. J. Petrol. Sci. Eng..

[28] Ji L, Li W, Shi W, Tian F, Agarwal R (2021). Effect of blade thickness on rotating stall of mixed-flow pump using entropy generation analysis. Energy.

[29] Ji L, Li W, Shi W, Chang H, Yang Z (2020). Energy characteristics of mixed-flow pump under different tip clearances based on entropy production analysis. Energy.

[30] Wu J, Zhou Y, Fan M (1999). A note on kinetic energy, dissipation and enstrophy. Phys. Fluids..

[31] Buxton ORH, Ganapathisubramani B (2010). Amplification of enstrophy in the far field of an axisymmetric turbulent jet. J. Fluid Mech..

[32] Benzi R, Biferale L, Calzavarini E, Lohse D, Toschi F (2009). Velocity-gradient statistics along particle trajectories in turbulent flows: The refined similarity hypothesis in the Lagrangian frame. Phys. Rev. E Stat. Nonlin. Soft Matter Phys..

[33] Varma AR, Ahmed U, Chakraborty N (1994). (2021) Effects of body forces on vorticity and enstrophy evolutions in turbulent premixed flames. Phys. fluids.

[34] Neamtu-Halic MM, Mollicone JP, van Reeuwijk M, Holzner M (2021). Role of vortical structures for enstrophy and scalar transport in flows with and without stable stratification. J. Turbul..

[35] Lin T (2021). Application of enstrophy dissipation to analyze energy loss in a centrifugal pump as turbine. Renew. Energ..

[36] Suh J (2018). Development of numerical Eulerian-Eulerian models for simulating multiphase pumps. J. Petrol. Sci. Eng..

[37] Roache PJ (1998). Verification of codes and calculations. AIAA J..

[38] Roache PJ (1994). Perspective: A method for uniform reporting of grid refinement studies. J. Fluid. Eng..

[39] Roache PJ (1997). Quantifucation uncerainty in computational fluid dynamics. Annu. Rev. Fluid. Mech..

[40] Kock F, Herwig H (2004). Local entropy production in turbulent shear flows: A high-Reynolds number model with wall functions. Int. J. Heat Mass Tran..

